# Long-Term Cultured Human Glioblastoma Multiforme Cells Demonstrate Increased Radiosensitivity and Senescence-Associated Secretory Phenotype in Response to Irradiation

**DOI:** 10.3390/ijms24032002

**Published:** 2023-01-19

**Authors:** Lina Alhaddad, Zain Nofal, Margarita Pustovalova, Andreyan N. Osipov, Sergey Leonov

**Affiliations:** 1 School of Biological and Medical Physics, Moscow Institute of Physics and Technology (National Research University), 141701 Dolgoprudny, Russia; 2 Department of Environmental Sciences, Faculty of Sciences, Damascus University, Damascus P.O. Box 30621, Syria; 3 State Research Center—Burnasyan Federal Medical Biophysical Center of Federal Medical Biological Agency (SRC-FMBC), 123098 Moscow, Russia; 4 N.N. Semenov Federal Research Center for Chemical Physics, Russian Academy of Sciences, 119991 Moscow, Russia; 5 Institute of Cell Biophysics, Russian Academy of Sciences, 142290 Pushchino, Russia

**Keywords:** glioblastoma multiforme, multinucleated giant cancer cell, senescent tumor cells (STC), stress-induced premature senescence (SIPS), senescence-associated secretory phenotype (SASP)

## Abstract

The overall effect of senescence on cancer progression and cancer cell resistance to X-ray radiation (IR) is still not fully understood and remains controversial. How to induce tumor cell senescence and which senescent cell characteristics will ensure the safest therapeutic strategy for cancer treatment are under extensive investigation. While the evidence for passage number-related effects on malignant primary cells or cell lines is compelling, much less is known about how the changes affect safety and Senescence-Associated Secretory Phenotype (SASP), both of which are needed for the senescence cell-based vaccine to be effective against cancer. The present study aimed to investigate the effects of repeated passaging on the biological (self-renewal capacity and radioresistance) and functional (senescence) characteristics of the different populations of short- and long-term passaging glioblastoma multiforme (GBM) cells responding to senescence-inducing DNA-damaging IR stress. For this purpose, we compared radiobiological effects of X-ray exposure on two isogenic human U87 cell lines: U87L, minimally cultured cells (<15 passages after obtaining from the ATCC) and U87H, long-term cultured cells (>3 years of continuous culturing after obtaining from the ATCC). U87L cells displayed IR dose-related changes in the signs of IR stress-induced premature senescence. These included an increase in the proportion of senescence-associated β-galactosidase (SA-β-Gal)-positive cells, and concomitant decrease in the proportion of Ki67-positive cells and metabolically active cells. However, reproductive survival of irradiated short-term cultured U87L cells was higher compared to long-term cultured U87H cells, as the clonogenic activity results demonstrated. In contrast, the irradiated long-term cultured U87H cells possessed dose-related increases in the proportion of multinucleated giant cancer cells (MGCCs), while demonstrating higher radiosensitivity (lower self-renewal) and a significantly reduced fraction of DNA-replicating cells compared to short-term cultured U87L cells. Conditioned culture medium from U87H cells induced a significant rise of SA-β-Gal staining in U87L cells in a paracrine manner suggesting inherent SASP. Our data suggested that low-dose irradiated long-term cultured GBM cells might be a safer candidate for a recently proposed senescence cell-based vaccine against cancer.

## 1. Introduction

Glioblastoma multiforme (GBM) is one of the most aggressive and most common malignant primary brain tumors in adults and often occurs in patients over 65 years of age [1,2]. Radiotherapy is still a treatment of choice for patients with GBM. However, its efficacy is limited by the dose that can be safely administered without eliciting serious side-effects, especially in elderly and debilitated patients, as well as the fact that recurrence, metastasis and radioresistance are common in GBM [3]. Radiotherapy can cause cancer cells to enter a state of stress-induced premature senescence (SIPS), thereby slowing down the cell cycle and further proliferation and allowing cancer cells to escape the DNA-damaging effects of X-rays. It is believed that SIPS rather than apoptosis, is preferentially induced in GBM cells after radio-and chemotherapy. Cancer cell senescence inhibits cancer growth by halting massive proliferation and increasing chances of immune clearance [4]. 

While the evidence for passage number-related effects on malignant primary cells or cell lines is compelling, much less is known about how the changes affect safety and SASP, both of which are needed for a recently proposed senescence cell-based vaccine to be effective against cancer. Indeed, it was discovered that senescent tumor cells induce a bigger immune response than dead cancer cells [5]. Inducing tumor formation in mice previously injected with senescent tumor cells led to the development of fewer tumors, with some developing none. Albeit the injection was less effective against pre-existing tumors, this research suggests that a potential senescent cell cancer vaccine could show promise.

The quality and the reproducibility of anti-cancer effects of senescence cell-based vaccines depends on the quality and safety of the tumor cells used to make the vaccine. In turn, these characteristics of tumor cells seem to depend not least on the degree of subcultivation of cancer cells. The effects of passage number are usually long lasting. Transformed and diseased cell lines are of special concern, since they represent abnormal starting populations. Over time, they may change both their genotypic and their phenotypic traits. In these cell types, one or all of the typical cellular checkpoint genes, such as p16/INK4a, pRB and p53, have been altered whereby the cells have become “eternal”. These alterations are often in parallel with other cellular mutations, and the continual subculture of these cell lines exacerbates genomic and phenotypic instability [6]. For example, reduced cancer stem cell characteristics in a side population (SP) of late-passage non-small cell lung cancer (NSCLC) cells was reported. These include increased frequency of tumor-initiating and self-renewal capacity, and resistance to DNA-damaging agent doxorubicin and ionizing radiation [7]. The discovery that the SP from long-term passage NSCLC cells was not consistently enriched for stem cell-like cancer cells in the cancer stem cell research field leads to the recommendation to use only low-passage cell lines.

Further complicating matters is that a passage level considered “high” for one cell line may not give rise to any significant passage effects in another. To prevent passage-related effects from affecting experiments, it is important to know how many experiments can be performed in a given set of cells at the same time, and how many there are.

How to induce tumor cell senescence to serve as a therapeutic strategy for cancer treatment is under extensive investigation. Thus, studies of the molecular mechanisms of cancer cell senescence and cancer cells’ escape from senescence will provide a new perspective on how cancer can be treated safely.

The overall effect of senescence on cancer progression and cancer cell resistance to X-ray radiation (IR) is still not fully understood and remains controversial. Senescence is a state where cells neither function normally nor die. Cells that are damaged or old may enter this suspended state. In this state, they do not reproduce, but they still communicate with the tumor microenvironment through SASP in a paracrine fashion [8]. Albeit stress-induced senescence is generally considered to be a tumor-suppressive mechanism [9], long-term treatment-induced senescence of cells may be harmful. Long-term induction of senescence will produce a tumor microenvironment that promotes inflammation and immunosuppression [10]. Hence, induction of cancer cell senescence as a recently suggested new therapeutic strategies against cancer [5,11,12,13] should be context-dependent and evaluated carefully based on the cancer age. Additionally, it is possible to develop new therapeutic strategies to combine priming (immunization) with IR-induced senescent patient-derived cancer cells with IR treatments [14].

Based on all these findings, we thought be worth investigating the effects of repeated passaging on the biological (self-renewal capacity and radioresistance) and functional (senescence) characteristics of different populations of short- and long-term cultivated GBM cells responding to senescence-inducing DNA-damaging IR stress. We compared U87 cell lines that differ significantly in the culture duration after receiving from the ATCC: low passage number U87 cell lines (U87L) that have undergone up to 15 passages and high passage number U87 cell lines (U87H) that have undergone more than 3 years of continuous passaging. Our results demonstrate that long-term cultivation may sensitize GBM cells to IR, while possessing the safest phenotype, including constitutive highest SASP and lowest self-renewal capacities. In contrast, the short-term cultivated GBM cells sustained higher reproductive survival possibly through an IR dose-related increase in the proportion of SA-β-Gal-positive cells (both MGCCs and non-MGCCs) that might ultimately lead to GBM radioresistance. 

## 2. Results

### 2.1. Long-Term Cultivation Leads to a Decrease in Clonogenic Growth of GBM Cell Line after Irradiation

**Figure 1:**
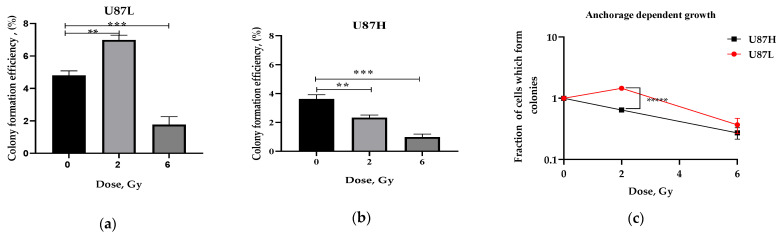

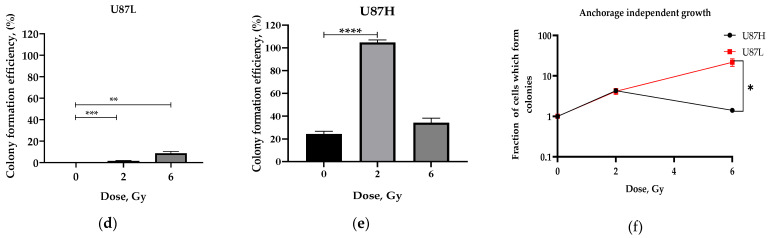
Plating efficiency of U87L (**a**) and U87H (**b**) cells after exposure to 2 and 6 Gy IR. Colony formation efficiency in soft agar of U87L (**d**) and U87H (**e**) cells after exposure to the same doses of IR. Reproductive survival of GBM cell lines was analyzed using survival fraction of cells grown under both anchorage-dependent (**c**) and anchorage-independent conditions (**f**). * *p* < 0.05; ** *p* < 0.01; *** *p* < 0.001; **** *p* < 0.0001; ***** *p* < 0.00001.

Clonogenic assay or colony formation assay is an in vitro cell survival assay based on the ability of a single adherent cell to grow into a colony and is a “gold standard” for assessing cancer cells’ radiosensitivity [15]. The number of colonies originating from single cells is expressed by plating efficiency (PE), an index which has emerged for normalization of surviving fractions based on the major premise that under untreated conditions the relation between the number of seeded cells and the number of resulting colonies is linear. PE is often used for determining the effects of growth factors and toxicity testing. We aimed to evaluate PE alteration of IR exposed minimally cultured (U87L) and long-term cultured (U87H) GBM cells. We observed that 2 Gy IR significantly increases PE of U87L cells (by 1.45 times, *p* < 0.01) and decreases its value after 6 Gy exposure (by 2.71 times, *p* < 0.001) compared to non-irradiated cells (Figure 1a). In contrast, U87H cells displayed a dose-dependent decrease in the PE by 1.55 times (*p* < 0.01) and by 3.67 times (*p* < 0.001) after irradiation at doses of 2 and 6 Gy, respectively (Figure 1b). Despite the PE results, U87L cells had higher survival fractions (SF) compared to U87H after irradiation at a dose of 6 Gy (*p* < 0.01) (Figure 1c), indicating their higher radioresistance growing under anchorage-dependent conditions. 

Anchorage-independent growth is the ability of transformed cells to grow independently of the attachment to a solid surface, and is a hallmark of carcinogenesis, anoikis resistance and propensity to tumor metastasis [16]. To evaluate whether the long-term culturing affects the anoikis resistance of two isogenic U87 cell sublines, we assessed their reproductive survival using anchorage-independent soft agar colony formation assay. Whereas U87L cells demonstrated increased ability to grow and to form colonies under non-adherent conditions (Figure 1d), the long-term cultured U87H cells had a significantly reduced ability (Figure 1e) both with and without IR exposure. Consequently, the survival fraction of U87L cells significantly exceeded that of U87H cells (Figure 1f) supporting the notion that long-term culturing leads to decreases in reproductive survival of the GBM U87H cell subline in response to IR-induced stress.

### 2.2. Influence of Preceding Cultivation Length on Metabolism of GBM Cells after Irradiation

**Figure 2:**
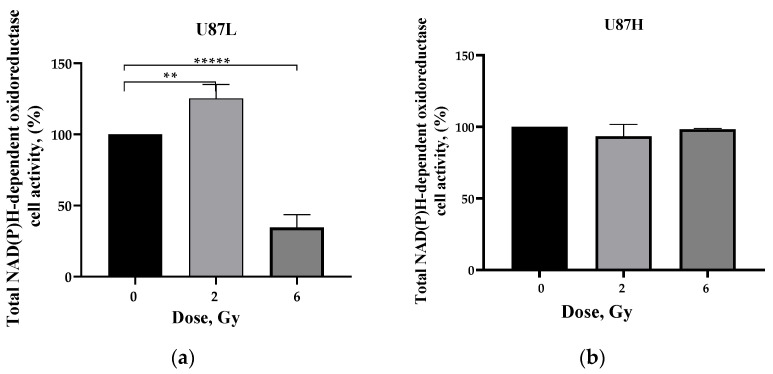
Assessment of the metabolic activity of U87L (**a**) and U87H (**b**) cells 7 days after irradiation at doses of 2 and 6 Gy using the Alamar Blue test in soft agar. ** *p* < 0.01; ***** *p* < 0.00001.

To understand the underlying mechanisms of the discovered clonogenic effect of long culturing of malignant cells, we evaluated the metabolic changes in cells of two U87 sublines forming the colonies in soft agar. Alamar Blue test has been widely used for cytotoxicity and viability assays based on the ability of NADPH oxidoreductases to reduce the oxidized, non-fluorescent, blue state of the compound to a fluorescent, pink state [17]. The significant increase (by 1.25 times, *p* < 0.01) and decrease by 2.83 times (*p* < 0.00001) in metabolic activity of U87L cells in response to IR at 2 and 6 Gy, respectively (Figure 2a), correlates well with the dynamics in their colony formation efficacy (Figure 1d) after IR exposure at the same doses. At the same time, irradiation did not lead to a change in metabolic activity of colony-forming long-term cultured cells of U87H subline (Figure 2b). Taken together, our results demonstrate that the retained ability of the U87L cell subline to modulate its own activity of NADPH oxidoreductases may underlie the high reproductive survival in response to IR-induced stress.

### 2.3. IR Increases the Proportion of Senescent MGCCs in the Short-Term, but Not in the Long-Term Cultivated GBM Cell Line 

**Figure 3:**
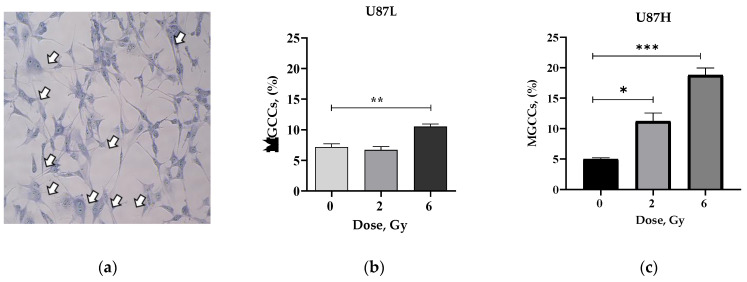

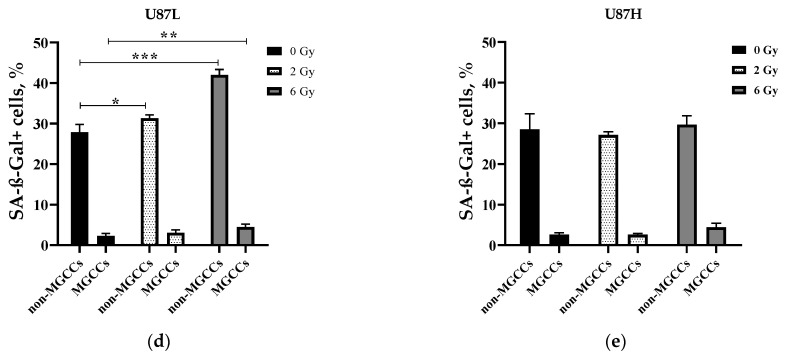
MGCCs formation in minimally-cultured and long-term cultivated GBM upon irradiation. Representative picture of MGCCs (indicated by arrows) (**a**). Change in the proportion (%) of MGCCs in U87L (**b**) and in U87H (**c**) cell lines 24 h after irradiation. Changes in the proportion (%) of SA-β-Gal+ populations in U87L (**d**) and U87H (**e**) cell lines 24 h after exposure to IR at doses of 2 and 6 Gy. * *p* < 0.05; ** *p* < 0.01, *** *p* < 0.001.

Advanced age is a major risk factor for the development of GBM [18]. At the same time, MGCCs arise in response to ionizing radiation in many tumors and contribute to cancer relapse by first entering a state of dormancy which is often accompanied by the stress-induced premature senescence (SIPS) phenotype and ultimately giving rise to cell progeny with stem-like properties [19]. We found that the proportion of MGCCs in response to irradiation at single therapeutically relevant doses of 2 and 6 Gy differs for U87 cell sublines depending on the duration of their preceding cultivation. We observed an increase in the proportion of MGCCs in U87L cells by 1.5 times (from 7.2% in non-irradiated cells to 10.5%, *p* = 0.006) 24 h after irradiation at a dose of 6 Gy (Figure 3b). For U87H cells, this effect was more pronounced: there was a statistically significant increase in the proportion of MGCCs by 2.2 times (from 5% in non-irradiated cells up to 11.2%, *p* = 0.0095) and 3.77 times (up to 18.8%, *p* = 0.0002) at 24 h after irradiation at doses of 2 and 6 Gy, respectively (Figure 3c).

As the stress-induced premature senescence (SIPS) phenotype is typically identified by senescence-associated β-galactosidase (SA-β-Gal) staining, we further aimed to compare the proportion of SA-β-Gal+ cells in the MGCCs and non-MGCCs populations of two GBM cell lines. 

The basal proportion of SA-β-Gal+ cells in the non-MNGCs population of non-irradiated U87H cells (27.9%) was almost the same as in the U87L cell line (28.5%) (Figure 3d,e). Ionizing irradiation of U87L cell lines caused significant increase in SA-β-Gal+ cells up to 31.3% (*p* = 0.07) and 42% (*p* = 0.0008) at 2 Gy and 6 Gy, respectively (Figure 3d). In contrast, the same populations of irradiated U87H cells did not change significantly, reaching 27.2% (*p* = 0.6) and 29.7% (*p* = 0, 7) at 2 Gy and 6 Gy, respectively (Figure 3e).

The proportion of SA-β-Gal+ MGCC population in U87L cell lines increased up to 3% (*p* = 0.2) and 4.5% (*p* = 0.014) in response to irradiation doses of 2 and 6 Gy (Figure 3d), while the proportion of SA-β-Gal+ MGCCs in the U87H cell line was statistically indistinguishable (Figure 3e). Consequently, the proportion of SA-β-Gal+ MGCCs in the U87L cell lines after irradiation increased from 31.5% (non-irradiated cells) up to 45% and 43% of total MGCC population irradiated at 2 Gy and 6 Gy, respectively. The SA-β-Gal+ MGCC proportion in U87H cell lines decreased from 52% (non-irradiated cells) down to 23% of total MGCC population after irradiation at doses of 2 and 6 Gy.

Thus, irradiation triggered an increase in the proportion of both SA-β-Gal-positive MGCCs and non-MGCC populations in U87L cell lines, leaving unchanged the level of this senescence marker in the U87H cell line.

### 2.4. Proliferative Activity of GBM Cells in Response to a Single Dose Irradiation

**Figure 4:**
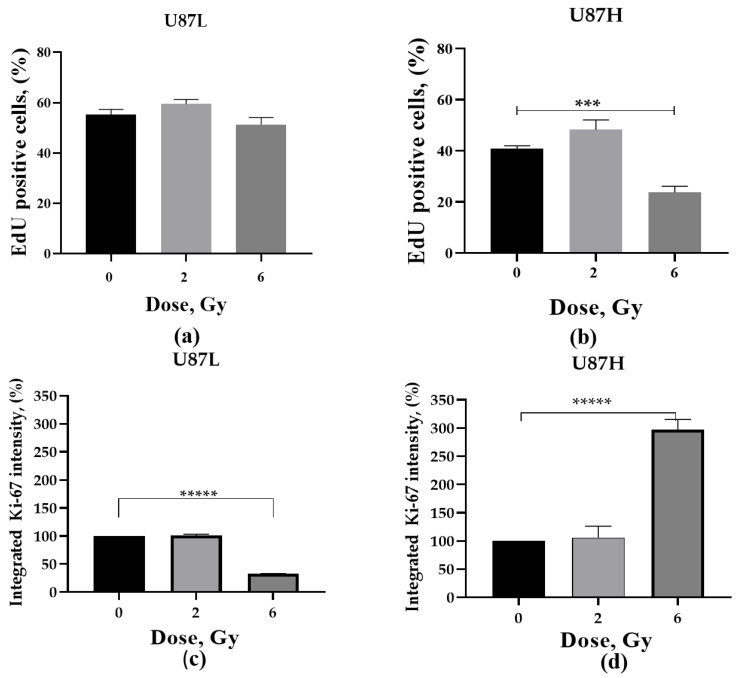
Assessment of the proliferative activity of minimally cultured U87L (**a**,**c**) and long-term cultured U87H cells (**b**,**d**). Change in the percentage of EdU+ cells (**a**,**b**), as well as change in the Ki-67 fluorescence intensity (%) (**c**,**d**) was measured 24 h after irradiation at doses of 2 and 6 Gy. *** *p* < 0.001; ***** *p* < 0.00001.

While eliminating tumor cells, cancer therapies could also induce a sustained proliferation arrest (treatment-induced dormancy) allowing a significant proportion of dormant cells to remain viable and metabolically active for long times post-treatment. We compared the level of EdU-positive (DNA replicating) cells between U87L and U87H lines using conventional Click-IT^TM^ EdU Alexa Fluor 488 cell proliferation assay. The irradiation did not affect the fraction of DNA-replicating (EdU+) U87L cells (Figure 4a), while it reduced the fraction of proliferating U87H cells (Figure 4b) after 6 Gy by 1.8 times (*p* < 0.001) compared to non-irradiated cells.

The Ki-67 protein is not required for cancer cell proliferation, but is required for all stages of carcinogenesis [20]. As seen from our data, 6 Gy irradiation caused completely opposite changes in Ki-67 expression: there was a more than 3 times increase (*p* < 0.0001) and almost 3 times decrease (*p* < 0.0001) in U87H (Figure 4d) and U87L (Figure 4c) cells, respectively. Thus, increased levels of Ki-67 expression led to an almost 2-fold decrease in the amount of DNA-replicating minimally-cultured U87H cells (Figure 4a), indicating inhibition of proliferation caused by an excess of Ki-67, which is in good agreement with the previous investigations [21]. On the other hand, a significant decrease in the percentage of Ki-67 in U87L cells may evidence a slowdown in their cell cycle without a significant change in the fraction of DNA-replicating cells (Figure 4a). 

### 2.5. Long-Term Cultured U87H Cells Demonstrate Senescence-Associated Secretory Phenotype

**Figure 5:**
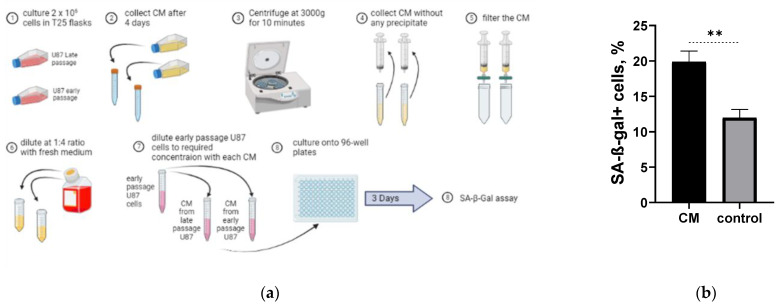
Assessment of the formation of SA-β-Gal positive cells in U87L line cultured in conditioned medium (CM) obtained from U87H cells. Schematic representation of the experiment (**a**). Percentage of SA-b-Gal positive cells formed in U87L cells cultured in CM and in the medium from U87L cell line (control) (**b**). ** *p* < 0.01.

To elucidate whether the changes in long-term cultivated U87H line are associated with Senescence-Associated Secretory Phenotype (SASP), we cultivated U87L cells with the condition medium (CM) obtained from U87H cells (Figure 5a). CM obtained from high passaged U87H cells increased the proportion of SA-β-Gal+ U87L cells by 1.6 times compared to control after incubation for 3 days (Figure 5b).

## 3. Discussion

Cellular senescence emerges in response to multiple extracellular or intracellular stress stimuli, such as telomeric dysfunction resulting from repeated cell division, mitochondrial impairment, oxidative stress, severe or irreparable DNA damage and chromatin disruption, and the expression of certain oncogenes. The senescence program causes permanent cell-cycle arrest, preventing the spread of damage to the next cell generation and precludes potential malignant transformation, which makes it a plausible anticancer mechanism during radiotherapy. However, this state of stable proliferative arrest is accompanied by the Senescence-Associated Secretory Phenotype (SASP), which entails the abundant secretion of pro-inflammatory molecules in the tissue microenvironment and contributes to age-related diseases, including GBM [22]. GBM is a primary brain tumor with a median age of diagnosis of 68–70 years [23]. The incidence rate of GBM increases with age from 1.25 per 100,000 people in adults of 35–44 years of age to 15.13 among older people of 75–84 years of age [24]. The basis for the increased incidence of GBM among elderly people remains poorly understood [18]. 

In the present study, we demonstrate the comparative analysis of X-ray irradiation exposure on two isogenic GBM cell lines: U87L, minimally cultured cells (<15 passages after obtaining from the ATCC) and U87H, long-term cultured cells (>3 years of continuous culturing after obtaining from the ATCC). During the cultivation period, U87H cells underwent several episodes of neosis—one of the forms of cell division, characterized by atypical karyokinesis through nuclear membrane budding, followed by asymmetric intracellular cytokinesis, producing various amounts of small mononuclear cells called Raju cells, which have an extended mitotic lifespan [25]. 

Short-term cultured U87L cells further demonstrated reduced radiosensitivity based on the resistance to anoikis assessed by soft agar assay (Figure 1f). Anoikis is the type of apoptotic cell death arising upon loss of attachment to the extracellular matrix and neighboring cells. Cancer cells may acquire resistance to anoikis, which allows them to survive after detachment from the primary sites, disseminate throughout the body and repopulate in secondary sites giving metastases [26]. Recent study demonstrated that clusters of circulating tumor cells were mostly large (≥5 µm), exhibited polyploidy and were associated with worse outcomes in non-small cell lung cancer (NSCLC) patients [27]. Here we demonstrate that the radioresistance of short-term cultured GBM cells may be associated with an increase in MGCC formation (Figure 3). 

Is the increase solely enough to confer radioresistance of short-term cultivated GBM cell lines? Corroborating the notion that MGCC formation in GBM induces senescence upon irradiation to sustain survival [28], we observed the increase of SA-β-Gal positive cells in U87L cell line. Surprisingly, despite the isogenic U87H cell line producing more MGCC (Figure 3c,b), there was no change in the fraction of SA-β-Gal+ cells in response to IR-induced stress (Figure 3d,e). 

To investigate the source of this discrepancy we analyzed the proportion of SA-β-gal-positive cells among MGCC and non-MGCC populations of both isogenic cell lines. In contrast to observations by Kaur et al. [28], we found almost the same significant amount of preexisting MGCC cells, while radiation-induced MGCC formation and senescent marker expression (SA-β-Gal) were different between isogenic cell lines (Figure 3). Indeed, we found for the first time that long-term cultivated cells lost the ability to produce significant amounts of SA-β-Gal+ cells within both MGCC and non-MGCC populations respectively to the doses of IR-induced stress. Thus, sustainably increased SA-β-Gal activity rather than a rise in total MGCC production might play a pivotal role in maintenance of the reproductive survival of the short-term cultivated U87L cell line in response to IR-induced stress. 

There are two phenotypes of cell dormancy: senescent cells (SIPS phenotype), in which cell cycle arrest is often irreversible, and resting cells (quiescence phenotype), which can be both non-proliferating, and slowly cycling [29]. While U87L cells demonstrated no change in DNA replication and decreased Ki-67 expression upon 6 Gy irradiation, U87H cells showed decreased DNA replicating activity accompanied by significant increase of Ki-67 expression (Figure 4). It is important to note that Ki-67 protein is not required for cancer cell proliferation, but rather required for all stages of carcinogenesis. The variability in Ki-67 expression is explained by its regulation through the cell cycle and is linked to heterochromatin packing density [20]. Our data corroborate previous findings that significant up-regulation of Ki-67 expression often down-regulates the DNA-replication in cancer cells [21]. Cancer treatment selects for Ki67– dormant cells, and at the same time, induces both apoptosis and tumor dormancy in Ki67+ tumor cells, resulting in an increased number of Ki67– and Ki67 low dormant cells [30]. Since SA-β-Gal activity can be found in both senescent and quiescent cells, in the present study we could not specify the precise dormancy phenotype of our GBM cell lines, albeit it warrants further investigation.

One of the hallmarks of the activation of cellular senescence is the secretion of a plethora of cytokines, chemokines, and growth factors, which is referred to as SASP [31]. It was demonstrated that oncogene-induced senescence acted to induce senescence in human primary melanocytes through an autocrine/paracrine fashion primarily by the secretion of IGFBP7 [32]. A senescent cell bystander effect was illustrated by Nelson et al., in which replicative senescent fibroblasts induce senescence in young fibroblasts in vitro through junction-mediated cell-cell contact [33]. Later on, it was shown that the senescence phenotype can be transmitted in a paracrine manner from fibroblasts undergoing oncogene-induced senescence to normal human fibroblasts and that the transmitted phenotype is stable [34]. In the present study, we observed that long-term culturing of cells alleviates SASP phenotype enabling induction of senescence marker in short-term cultured U87L cells (Figure 5b). The last, not least, our findings might have important clinical implications. The recent discovery that senescent tumor cells induce a bigger immune response than dead cancer cells [5] suggests that a potential senescent cell cancer vaccine could show promise. It is possible to develop new therapeutic strategies to combine priming (immunization) with IR-induced senescent patient-derived cancer cells with IR treatments [14].

Moreover, other recent studies have also shown that in situ-induced senescent cancer cells cause the adaptive immune system to become active. These cells can be caused by either GATA6 [35] or ZNF24 [12,13] ectopic expressions. Alternatively, cancer cell senescence can be alleviated by HMGCR inhibitor statins [36]. This offers a new strategy for the treatment of pancreatic cancer by suppression of up-stream TFCP2 signaling and induction of senescence. 

Now, the question of how senescent cells interact with the adaptive immune system has become a subject of intensive studies. The immune system activation includes dendritic cells and CD8 T-cells recruited by senescent cancer cells producing SASP [37]. These effects were supplemented by inducing the processing and presentation of MHC-I antigens elicited by either X-ray irradiation [38] or doxorubicin [5]. Moreover, senescence related-genes controlling immune cell infiltration of senescence will produce a tumor microenvironment and responses to immune checkpoint blockage (ICB) as a lung cancer therapy [11]. 

While the evidence for passage number-related effects on malignant primary cells or cell lines is compelling, much less is known about how the changes affect safety and SASP, both of which are needed for the senescence cell-based vaccine to be effective against cancer. In this regard, with some assumptions and reservations, it is possible to consider the U87H cell line as an “aging” subline of U87L cells. Then, short-term cultured U87L cells demonstrated higher reproductive survival compared to their isogenic “aged” counterpart after X-ray radiation exposure. Thus, long-term cultivated GBM cells possess the safest phenotype: highest sensitivity to IR and constitutive highest SASP, while having the lowest self-renewal capacities. Our data provides novel molecular insights into a multistep process of post-radiation reproductive survival in GBM and can be exploited for age-related anti-cancer interventions in the treatment of GBM.

## 4. Materials and Methods 

### 4.1. Cell Culture

The ATCC human U87 GBM cell lines were used in our study. U87 cell lines were cultured in DMEM (Gibco, Thermo Fisher Scientific, Waltham, MA, USA) supplemented with 10% FBS (BioloT, Saint Petersburg, Russia), 1% L-glutamine (Gibco, Grand Island, NY, USA), and 1% antibiotics (100 U/mL penicillin, 100 μg/mL streptomycin) (Sigma-Aldrich, St. Louis, MO, USA). U87L cell lines, which have undergone (up to 15 passages) after thawing and culturing of the original ampoule from ATCC, and the U87H cell lines, which have undergone long-term continuous cultivation (more than 3 years) were used. Cell lines were kept in a humidified atmosphere with 5% CO_2_. 

### 4.2. Irradiation

Cells were irradiated at room temperature in doses of 2 and 6 Gy using a 200 kV X-ray RUB RUST-M1 biological unit (Ruselectronics, Moscow, Russia). The dose rate was 0.85 Gy/min ± 10%.

### 4.3. Anchorage-Dependent Growth Assay

After reaching 70–80% confluence, the cells were exposed to X-ray radiation at doses of 2 and 6 Gy. Immediately after irradiation, control cells and cells irradiated at doses of 2 and 6 Gy were harvested using a 0.05% solution of trypsin-EDTA (PanEco, Moscow, Russia) and seeded on Petri dishes (60 mm in diameter) at concentrations of 5 × 10^2^, 8 × 10^2^ and 1 × 10^3^ cells/dish, respectively. Then, Petri dishes were incubated at 37 °C in a humidified atmosphere with 5% CO_2_ for 14 days to form colonies. After that, the culture medium was removed by aspiration from each dish, the cells were fixed with 100% methanol for 15 min at room temperature, followed by Giemsa staining for 15 min. Only colonies containing ≥50 individual cells were counted manually.

Plating Efficiency (PE) and Fraction of Cells, which form Colonies (FCC), were calculated using the following equations:

PE = # of colonies formed/# of cells seeded × 100%(1)

FCC = # of colonies formed after irradiation/[# of cells seeded × PE](2)

### 4.4. Soft Agar Clonogenic Assay 

6-well plates were coated with 0.6% agar-agar (Sigma-Aldrich, St. Louis, MO, USA) pre-warmed to 43 °C in complete medium. Immediately after irradiation, cells were collected and cell concentrations were adjusted to 5 × 10^2^, 8 × 10^2^ and 1 × 10^3^ cells/mL. Cell/0.3% noble agar mixtures were added into each well and allowed to solidify for another 30 min at room temperature before placing into a 37 °C humidified cell culture incubator. Three weeks later, colonies were stained with 5% Crystal Violet solution and their number was counted manually.

### 4.5. AlamarBlue Cell Viability Assay in Soft Agar

100 µL of 0.3% agar-agar in growth medium was added to a 96-well microculture plate (Sigma-Aldrich, TPP Zellkultur. Testplatte, Trazadingen, Switzerland) and left to solidify at room temperature for 30 min. Immediately after irradiation, cell suspensions were gently mixed with 0.2% agar-agar preheated on water at 43 °C. The final concentration of cells per well was 6 × 10^2^ cells/100 µL. Immediately after solidification, 50 µL of 0.3% agar-agar in growth medium was added to each well as a feeding layer and allowed to solidify for 30 min at room temperature. Cells were incubated under standard CO_2_ incubator conditions (5% CO_2_, 37 C) for 7 days, after which 10% AlamarBlue reagent (Invitrogen, Frederick, MD, USA) was added to each well and cells were incubated for 2 h at 5% CO_2_ and 37 °C. The fluorescence of the AlamarBlue reagent was determined using a CLARIOstar instrument (BMG LABTECH, Ortenberg, Germany) at excitation/emission wavelengths of 530/590 nm. The obtained data were processed using the MARS data analysis software (BMG LABTECH, Ortenberg, Germany).

### 4.6. Wright-Giemsa Staining and Analysis of MGCCs

Cells were cultured in a 96-well plate (Sigma-Aldrich, TPP Zellkultur. Testplatte, Trazadingen, Switzerland) at a concentration of 5 × 10^3^ cells/0.32 cm^2^. 24 h after irradiation, the cells were fixed with absolute methanol for 5 min, after which they were stained using Wright-Giemsa solution diluted 1:10 in 1X PBS (pH 6.6) for 1 h, followed by washing with 1X PBS (pH 6.6) and distilled water. The average number of MGCCs was calculated in five microscopic fields of view at ×100 magnification.

### 4.7. Senescence Associated β Galactosidase Assay (SA-β-Gal)

Detection of the activity of aging-associated beta-galactosidase (SA-β-Gal) (a marker of the cellular senescence phenotype) was performed using a commercial cell senescence assay kit (EMD Millipore KAA002, CA, USA). The presence of the β-Gal enzyme in cells is determined by the characteristic green staining of the cytoplasm. The cells were stained according to supplemented manufacturer protocol with the following modification: at the final 1X PBS washing step, the cell nuclei were stained with 1 μg/mL Hoechst 33342 (Thermo Scientific, Rockford, IL, USA). Such modification significantly improves the quality of counting of SA-β-Gal-negative cells [39,40]. The stained cells were visualized using an EVOS^®^ FL Auto Imaging System (Fisher Scientific, Pittsburgh, PA, USA) with 20× objective. The proportion of SA-β-Gal-positive cells was calculated manually.

### 4.8. Click-iT™ EdU Alexa Fluor 488 (Cell Proliferation Assay)

The Click-iTTM EdU Alexa Fluor 488 Imaging kit (Invitrogen, Thermo Fisher Scientific, Waltham, MA, USA) was used to assess cell proliferation. Cells were visualized using an ImageXpress Micro XL automated digital imaging and High Content imaging and Analysis system (Molecular Devices LLC, San Jose, CA, USA).

### 4.9. Immunofluorescence Analysis of Ki-67 

Cells (at a concentration of 2 × 10^3^ cells/0.05 cm^2^) were seeded in a 384-well plate for 24 h. Then, cells were washed briefly in 1X PBS (pH 7.4) and fixed with 4% formaldehyde for 15 min, followed by 3 rinses in 1X PBS (pH 7.4). After blocking cells with 6% BSA (bovine serum albumin) in 1X PBS (pH 7.4) for 1 h at room temperature, cells were incubated with mouse monoclonal Ki-67 antibody (5 μg/mL, clone Ki-S5, Sigma-Aldrich, Darmstadt, Germany) diluted in 1X PBS with 1% BSA and 0.3 % TritonX-100 for 1 h at room temperature. After 3 rinses in PBS, cells were incubated for 1 h at room temperature with Alexa Fluor 555 goat Anti-mouse secondary antibody (1:500, Merck-Millipore, Burlington, VT, USA) diluted in 1X PBS with 1 % BSA and 0.3 % Triton X-100. Nuclei were counterstained with Hoechst 33342 Solution (dilution 6 μg/mL, Thermo Scientific, Rockford, IL, USA). Cells were imaged and inner integrated fluorescence intensities of cells were calculated using the ImageXpress XL fluorescence microscopy (Molecular Devices LLC, San Jose, CA, USA).

### 4.10. Conditioned Medium Experiment

U87H and U87L cells were cultured in T25 flasks with 2 × 10^6^ cells in each and incubated at 37 °C and 5% CO_2_. The conditioned medium from each flask was collected on the 4th day of incubation and centrifuged at 3000× *g* for 10 min; then, without collecting any debris from the bottom of the tubes, the conditioned medium from each flask was filtered through 0.22 mm filter and diluted in 1:4 ratio with fresh DMEM medium. U87L cells were later cultured in 6 repeats with the conditioned medium form U87H cells and 6 repeats with the conditioned medium from U87L cells into 96-well plate with seeding density of 3125 cell/cm^2^. After 3 days of incubation SA-β-Gal assay was performed.

### 4.11. Statistical Analysis

Statistical data processing was carried out using statistical software GraphPad Prism 9.0.2.161 (GraphPad Software, San Diego, CA, USA) and EXCEL 2010 Software (Microsoft, Redmond, WA, USA). Data presented as means ± SEM. Significance levels are marked with asterisks: * *p* < 0.05, ** *p* < 0.01, *** *p* < 0.001, **** *p* < 0.0001, ***** *p* < 0.00001.

## Data Availability

Not applicable.
